# Spatiotemporal Angiogenic Patterns in the Development of the Mouse Fetal Blood–Brain Barrier System During Pregnancy

**DOI:** 10.3390/ijms26083862

**Published:** 2025-04-18

**Authors:** Samuel Nofsinger Brown, Philemon Shallie, Connor A. Sierra, Neha Nayak, Anthony O. Odibo, Paula Monaghan-Nichols, Nihar R. Nayak

**Affiliations:** 1 Department of Obstetrics and Gynecology, University of Missouri Kansas City School of Medicine, Kansas, MO 64108, USA; srbnc5@health.missouri.edu (S.N.B.); pdsxfn@umkc.edu (P.S.); nehan77@gmail.com (N.N.); aoodibo@umkc.edu (A.O.O.); 2 University of Missouri School of Medicine, Columbia, MO 65212, USA; casyxw@health.missouri.edu; 3 Department of Biomedical Sciences, University of Missouri Kansas City School of Medicine, Kansas, MO 64108, USA; nicholsap@umkc.edu

**Keywords:** blood–brain barrier, development, fetus, pericytes, endothelial, tight junction, angiogenesis, pregnancy

## Abstract

Understanding the timing of fetal brain vulnerability to inflammatory changes in pregnancy complications is crucial for predicting neurodevelopmental risks. Beyond the placenta, the developing brain’s vascular system is believed to form a secondary defense, the blood–brain barrier (BBB), which restricts harmful substances that could disrupt neurodevelopment. However, the precise timing and mechanisms underlying BBB development are poorly understood. In this study, we examined the spatiotemporal expression of key BBB components and fetal brain vascularization in mice from gestational days (GD) 10 to 18. Fetal brain sections were immunostained to identify BBB components, including CD31, Factor VIII, NG2, and claudin-5. Our results showed that endothelial precursor cells form the primitive vascular network in a caudal-to-rostral gradient by GD10, with pericyte recruitment stabilizing vessels by GD12 in a lateral-to-medial gradient that aligns with neurogenesis, despite some regional exceptions. However, Factor VIII was not detected until GD15, and claudin-5 until GD18, suggesting a significant delay in endothelial maturation and tight junction formation. These findings highlight the critical timing of structural developments in the fetal brain vasculature and its vulnerability to placental diseases, laying the groundwork for future research on the impact of placental disorders on fetal brain development and potential therapeutic interventions.

## 1. Introduction

During fetal development, the placenta plays a critical role in regulating the exchange of gases, nutrients, and waste between the mother and fetus, while also serving as a protective barrier that shields the developing fetal organs from potentially harmful agents [1,2]. In addition to the placenta, the developing vascular system in the brain is thought to establish a secondary line of defense, safeguarding the fetal brain by restricting the entry of harmful substances that could impede neurodevelopment [1,3]. This protective mechanism, known as the blood–brain barrier (BBB), is formed by complex interactions between endothelial cells, pericytes, and neurons [4,5,6,7]. These interactions are believed to stabilize the vasculature and occur across distinct spatial compartments of the developing brain, which is essential for maintaining the homeostasis required for normal neural growth [4,6,7]. Recent studies suggest that the BBB begins to form during embryogenesis and continues through early postnatal periods, prior to the generation of astrocytes [4]. However, the interactions between endothelial cells and other developing brain cells in various compartments, as well as the precise timing of the formation of different key components of the BBB, are not yet fully understood [4,8,9,10].

Understanding the mechanisms that protect the developing brain is crucial for clinical outcomes, as disruptions in this process are believed to result in significant neurodevelopmental impairments [11]. Such impairments are often associated with pregnancy complications, particularly when placental function is compromised, which can interfere with brain vascularization and the formation of the BBB [12,13]. Conditions like preeclampsia, fetal growth restriction (FGR), and gestational diabetes have been linked to neurodevelopmental disorders, potentially due to abnormalities in BBB formation and resulting neuroinflammation [11,14]. Moreover, various harmful molecules, including certain medications used during pregnancy, inflammatory mediators, and environmental toxins, can bypass the placental barrier and reach the fetus [15,16]. Even small exposures to these molecules over the course of gestation can result in cumulative harm to the fetal brain [16]. Although studies have shown that the recruitment of pericytes and the establishment of tight junction impermeability occur early in brain development, periods of heightened sensitivity and vulnerability to toxins still need to be better understood [4,17,18]. These critical windows of susceptibility depend on the specific developmental processes occurring at different stages of gestation and remain poorly characterized [16].

In the adult brain capillaries, the BBB is primarily composed of microvascular endothelial cells on the inside that are tightly connected by a network of tight junctions, and on the outside supported by pericytes, astrocytes, and specialized extracellular matrix, which together contribute to the barrier’s integrity and functionality [18]. Recent research has greatly advanced our understanding of BBB development and functions at the molecular and cellular levels [19,20]. Evidence from morphological, molecular, and barrier function studies has demonstrated that key components of the BBB are formed during the fetal and early postnatal periods, and BBB function starts early during fetal development [4,20]. However, the coordinated expression and regulation of these molecules across various stages of fetal development, and their relationship to neuronal maturation, have not been fully explored [21]. It is known that endothelial precursor cells initially form a primitive vascular network, which becomes more refined and stabilized as pericytes are recruited and tight junctions are established [4]. Yet, the developmental timeline of structural developments in the fetal brain vasculature in the context of BBB formation and its regulation by neuronal development remains unclear [21].

In this study, we investigated the spatiotemporal expression of critical BBB molecules during fetal brain development at key gestational stages (GD10, GD12, GD15, and GD18), aiming to assess their potential role in the early formation of the fetal BBB. Our findings provide new insights into the intricate relationship between vascular development, neurogenesis, and BBB formation in the early fetal brain. We highlight the timing and coordination of these processes, shedding light on potential vulnerabilities in response to placental dysfunction.

## 2. Results and Discussion

The placenta plays a crucial role in protecting the fetus by acting as a barrier against harmful agents, with its protective function thought to be further reinforced by a secondary barrier mechanism in the fetal brain—the fetal blood–brain barrier (BBB). While the precise timing of BBB formation remains debated, recent studies have provided compelling evidence of early morphological and functional characteristics of barrier activity in the developing fetal brain in both humans and rodents [18,22]. In this study, we examined the spatiotemporal expression of key barrier molecules from the early stages of angiogenesis and perineural vascular plexus formation during fetal brain development at critical gestational stages (GD10, GD12, GD15, and GD18). These findings highlight the coordinated expression of these molecules and their relationship to neuronal maturation across different gestational periods.

**Figure 1:**
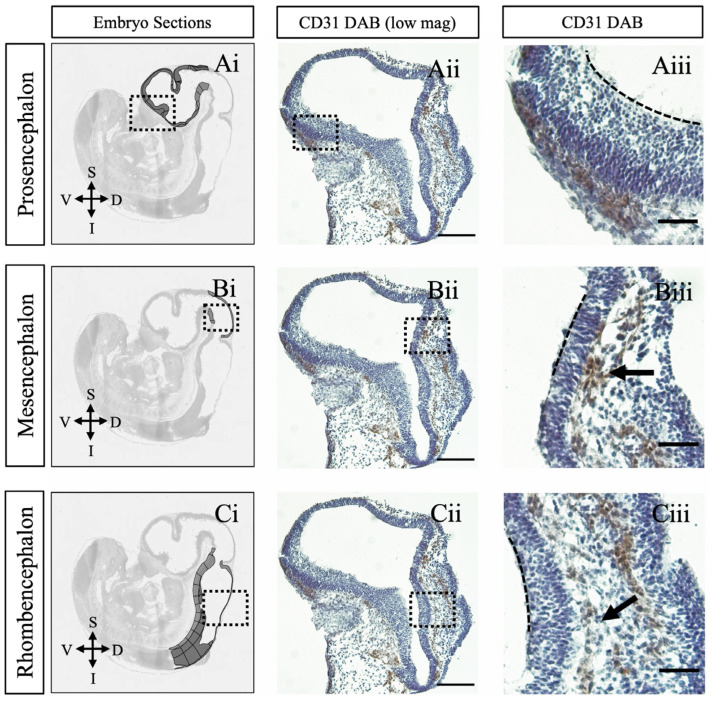
Endothelial precursor cells encompass the neural tube by GD10. Sagittal sections of mouse brain at GD10 immunostained for endothelial cells with CD31. (**Ai**–**Ci**) Representative anatomical images from the Allen Developing Mouse Brain Reference Atlas (https://atlas.brain-map.org, accessed on 23 February 2025) [26]. (**Aii**–**Cii**) Low magnification views of highlighted brain regions in (**Ai**–**Ci**). (**Aiii**–**Ciii**) High magnification views of boxed regions in (**Aii**–**Cii**). The perineural vascular plexus (PNVP) encircles the entire brain surface, with a particular emphasis on the alar and caudal regions of the neural tube. Perforating vessels converge to form the subventricular plexus (SVP) within the mesencephalon and rhombencephalon (indicated by arrows), demonstrating a caudal-to-rostral progression in SVP formation. The dashed line marks the boundary between the ventricular lumen and the ventricular surface of brain tissue. GD = embryonic day. Scale bars: (**Aii**–**Cii**) 300 μm, (**Aiii**–**Ciii**) 1000 μm.

We investigated the sequence of vascularization and BBB component assembly in the developing fetal brain. Embryonic neural tube sections at GD10 (embryonic day 9.5) were immunostained for CD31, factor VIII, NG2, and claudin-5. Our findings show that CD31-positive endothelial precursor cells (EPCs) formed around the neural tube in a caudal-to-rostral pattern, giving rise to the perineural vascular plexus (PNVP), particularly in the alar regions (Figure 1). However, NG2, Factor VIII, and claudin-5 were not detected at this early stage. We also observed the formation of the subventricular plexus (SVP) in the mesencephalon and rhombencephalon, following the same caudal-to-rostral progression (Figure 1(Biii,Ciii)). This supports a caudal-to-rostral gradient in SVP development. While some studies have suggested that PNVP formation follows a random pattern with no distinct spatial organization implicating heterochrony or prosomeric mapping [4,10,23,24], our observations are consistent with earlier findings regarding the temporal progression of PNVP formation in mice [25] and the caudal-to-rostral gradient observed in rats [4].

**Figure 2:**
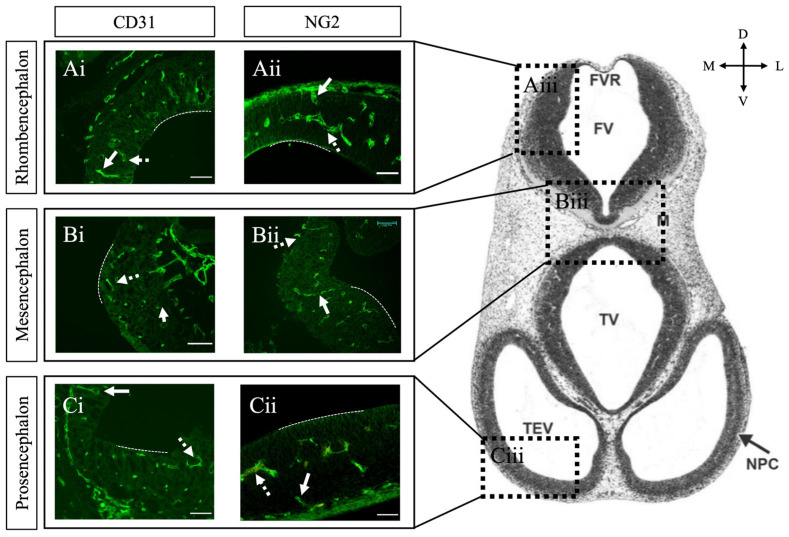
Pericytes invade the brain parenchyma by GD12. Transverse tissue sections of mouse brain at GD12 immunostained for endothelial cells with CD31 and pericytes with NG2. (**Ai**–**Ci**) CD31 immunostaining in developing rhombencephalon, mesencephalon, and prosencephalon. (**Aii**–**Cii**) NG2 immunostaining in developing rhombencephalon, mesencephalon, and prosencephalon. (**Aiii**–**Ciii**) Representative anatomical image of corresponding areas of analysis (**Ai**–**Cii**) reproduced with permission from Chen et al. 2017 [32]. Pericytes are associated with vessels of the PNVP, perforating radial vessels (solid arrows), and SVP (dashed arrows) across all caudal and rostral regions. The dashed line indicates the ventricular surface of brain tissue. GD = gestational day. E = neuroepithelium. FV = fourth ventricle. FVR = fourth ventricle roof. M = mesenchyme. NPC = neopallial cortex. TEV = telencephalic vesicle. TV = third ventricle. Scale bar 50 μm.

**Figure 3:**
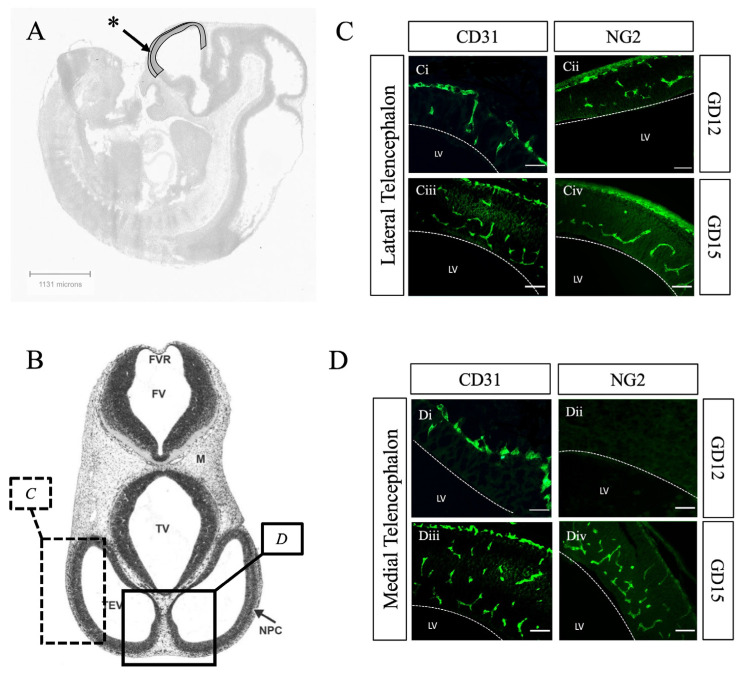
Pericytes are recruited to endothelial cells of the telencephalon in a lateral to medial gradient. (**A**) Representative sagittal anatomical images of a GD12 mouse embryo denoting the telencephalon * from the Allen Developing Mouse Brain Reference Atlas (https://atlas.brain-map.org, accessed on 23 February 2025) [26]. (**B**) Representative transverse anatomical image of GD12 mouse neural tube, with marked regions corresponding to the lateral telencephalon (*C*) and medial telencephalon (*D*). Reproduced with permission from Chen et al. 2017 [32]. (**C**) Transverse section of GD12 and GD15 lateral telencephalon immunostained for endothelial cells with CD31 (**Ci**,**Ciii**) and pericytes with NG2 (**Cii**,**Civ**). (**D**) Transverse section of GD12 and GD15 medial telencephalon immunostained for endothelial cells with CD31 (**Di**,**Diii**) and pericytes with NG2 (**Dii**,**Div**). In the lateral telencephalon, pericytes are recruited to PNVP and SVP vessels at GD12 and GD15. In contrast, PNVP and SVP vessels of the medial telencephalon (**D**) lack pericyte coverage at GD12 but show pericyte recruitment by GD15. These results highlight the lateral to medial gradient of pericyte recruitment within the telencephalon. GD = gestational day. FV = fourth ventricle. FVR = fourth ventricle roof. M = mesenchyme. NPC = neopallial cortex. TEV = telencephalic vesicle. TV = third ventricle. LV = lateral ventricle. Scale bar 50 μm.

By GD12, we observed that CD31-positive endothelial cells had infiltrated the neuroepithelium, forming a robust SVP across all three major brain regions (Figure 2(Ai–Ci)). These findings align with previous studies on SVP formation between GD11 and GD12 [4,27,28,29]. We observed no difference in the extent of SVP formation between brain regions at this time point. While Daneman et al. did not comment on SVP patterning, they identified a caudal-to-rostral gradient to radial vessel formation in the rat brain [4]. Differences in species and windows of observation may reflect differences in timing and recruitment compared to our study. At this stage, we did not detect Factor VIII or claudin-5 expression. However, NG2-positive pericytes were extensively recruited to both the PNVP and SVP in the prosencephalon, mesencephalon, and rhombencephalon by GD12 (Figure 2(Aii–Cii)). Notably, the developing telencephalon showed a higher density of NG2-positive pericytes associated with lateral vessels compared to medial vessels (Figure 3), suggesting a lateral-to-medial gradient of pericyte recruitment. This gradient seems to align with the pattern of neurogenesis in the developing cortex and is consistent with previous studies on pericyte recruitment to cortical vessels [27,30] and a dextran permeability assay by Ben Zvi et al. demonstrating that lateral cortical vessels are relatively more impermeable at GD14 compared to medial vessels [20]. Given that pericyte recruitment to CNS vessels is linked to the onset of barrier formation [4], and the establishment of BBB integrity [31], these results collectively suggest that a critical step in fetal BBB development occurs at this gestational stage.

**Figure 4:**
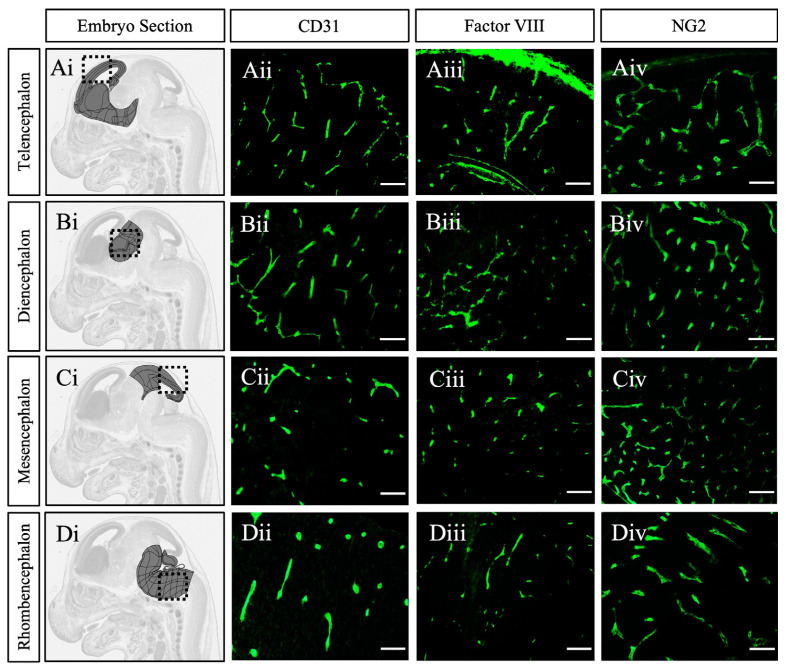
Endothelial cells express Factor VIII by GD15. (**Ai**–**Di**) Representative anatomical images of corresponding areas of analysis (highlighted) from the Allen Developing Mouse Brain Reference Atlas (https://atlas.brain-map.org, accessed on 23 February 2025) [26]. Sagittal tissue sections of GD15 mouse telencephalon, diencephalon, mesencephalon, rhombencephalon immunostained for endothelial cells with CD31 (**Aii**–**Dii**), Factor VIII (**Aiii**–**Diii**), and pericytes with NG2 (**Aiv**–**Div**). Factor VIII is uniformly expressed across all regions, coinciding with positive CD31 and NG2 signals, which demonstrates that the vessels have adopted a mature phenotype by GD15. GD = gestational day. Scale bar 50 μm.

**Figure 5:**
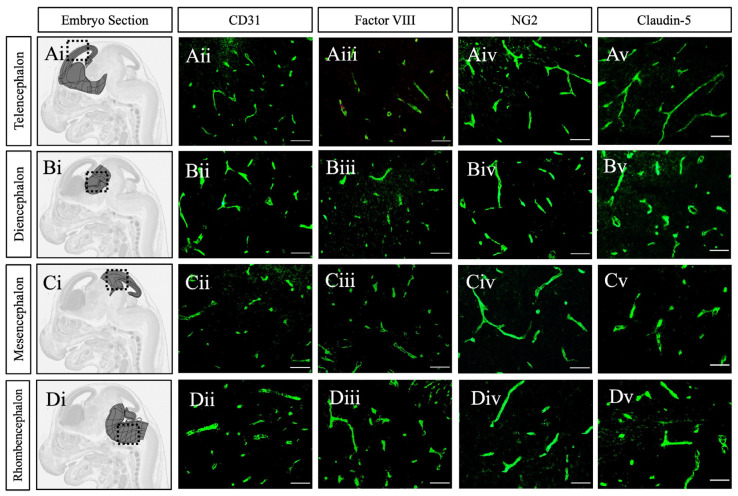
Endothelial cells express tight junctions by GD18. (**Ai**–**Di**) Representative anatomical images of corresponding areas of analysis (highlighted) from the Allen Developing Mouse Brain Reference Atlas (https://atlas.brain-map.org, accessed on 23 February 2025) [26]. Sagittal tissue sections of GD18 mouse telencephalon, diencephalon, mesencephalon, rhombencephalon immunostained for endothelial cells with CD31 (**Aii**–**Dii**), Factor VIII (**Aiii**–**Diii**), pericytes with NG2 (**Aiv**–**Div**), and tight junctions with claudin-5 (**Av**–**Dv**). In addition to CD31, NG2, and Factor VIII, Claudin-5 is uniformly expressed across all regions, indicating that mature endothelial cells express tight junctions by GD18. GD = gestational day. Scale bar 100 μm.

By GD15, all CD31-positive endothelial cells in vessels across various brain regions uniformly expressed Factor VIII, with no observable gradient (Figure 4). Within the telencephalon, NG2-positive pericytes were uniformly distributed along both lateral and medial vessels, suggesting a delay in pericyte recruitment to the medial vessels (Figure 3). The consistent expression of Factor VIII, along with robust pericyte recruitment in all vessels at this stage, indicates the completion of a key step in vascular maturation and the formation of the fetal blood–brain barrier (BBB). This observation aligns with previous reports showing Factor VIII expression after GD13 [33] and the onset of functional impermeability in cerebral vessels by GD16 [20]. However, there remains some controversy regarding the temporal expression of claudin-5. Our results did not detect reliable claudin-5 signals at GD15, but in addition to the consistently high expression of CD31, Factor VIII, and NG2, we observed strong claudin-5 expression in vessels at GD18 (Figure 5). This finding contrasts with some earlier studies that reported early claudin-5 detection, though there is considerable variability in the timing of detection across reports. Differences in methodologies, including the use of RNA versus protein assays, different reagents, and varying brain regions analyzed, likely contribute to this inconsistency [4,9,10,20]. Despite this, our findings—supported by functional permeability assays from other studies [9,19,20]—suggest that tight junctions are formed in the vessels after GD15, marking one of the final stages in the development of the fetal BBB.

**Figure 6:**
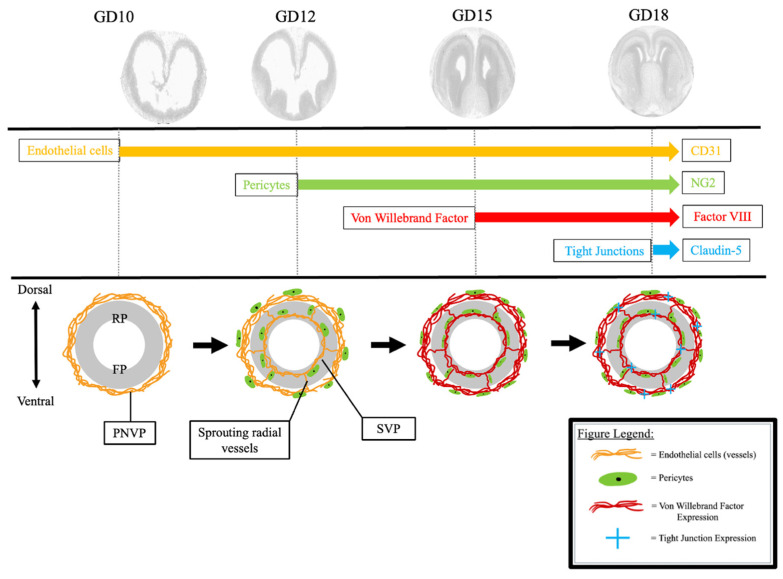
Pregnancy stage-specific sequential acquisition of BBB components in the fetal brain vessels. Endothelial precursor cells assemble into the PNVP to encompass the superficial neural tube by GD10. Radial vessels sprout from the PNVP and extend toward the ventricle of the neural tube, where they grow perpendicularly to form the SVP by GD12. Pericytes are recruited to the vessels of the SVP and PNVP by GD12. At GD15, Factor VIII is expressed in all endothelial cells of both the SVP and PNVP, suggesting that the vessels have adopted a mature phenotype by GD15. Finally, by GD18 the endothelial cells express tight junctions within the SVP and PNVP. Representative anatomical images of mouse brain from the Allen Developing Mouse Brain Reference Atlas (https://atlas.brain-map.org, accessed on 23 February 2025) [26]. PNVP = perineural vascular plexus. SVP = subventricular plexus. RP = rostral plate. FP = floor plate.

While several studies have investigated the expression of individual key components of the BBB during fetal brain development in mice, there is considerable variation in the reported timing of expression. These discrepancies are largely due to differences in methodologies, reagents, and the specific brain regions analyzed. Notably, no prior study has provided a comprehensive examination of multiple BBB components within the same animal model across the entirety of pregnancy [4,9,10,20]. Our study reveals the precisely coordinated expression of key BBB molecules across various regions of the fetal brain at critical developmental stages, in alignment with neuronal maturation. This process initiates with the formation of the primitive vascular network from endothelial precursor cells at GD10, followed by the recruitment of pericytes to stabilize these nascent vessels by GD12. By GD15, the vessels mature, and the establishment of tight junctions occurs in the later stages of gestation, after GD15 (Figure 6).

Our findings have several important clinical implications. The delayed maturation of vessels in the medial telencephalon, the precursor to the hippocampus, as observed in our study, may make this region susceptible to adverse conditions during early gestation. Hippocampal defects are closely linked to neurodevelopmental and learning disorders [34]. Furthermore, maternal immune activation prior to GD15 has been shown to disrupt hippocampal morphology, impair synaptic function, and delay myelination and axonal development [35,36]. Additionally, Factor VIII, which is crucial for maintaining vascular integrity and exhibits anti-inflammatory properties, plays a key role in BBB stability. Factor VIII-deficient mice exhibit increased BBB permeability and greater susceptibility to BBB breakdown under inflammatory conditions [37,38]. Our findings emphasize the expression of Factor VIII and claudin-5 during the later stages of pregnancy, indicating that the fetal brain continues to be vulnerable to placental diseases related to inflammation and oxidative stress, including preeclampsia and preterm birth, even in these later stages of gestation [16,39]. However, while our data from the mouse model provide valuable insights, caution should be exercised when extrapolating these findings to human fetal neurodevelopment and neurological diseases, as there are several human-specific differences in neuronal development, including variations in cortical expansion, complexity of neuronal subtypes, developmental timelines, cellular interactions, and distinct gene expression and regulation patterns [40,41].

In sum, this study highlights the critical timing of vascular developments in the fetal brain and its vulnerability to pregnancy complications. Our findings emphasize this timing in neurodevelopmental vulnerability, which could inform the identification of optimal windows for therapeutic intervention. We underscore the urgent need for a deeper understanding of the etiology of pregnancy complications and the development of biomarkers for their early detection. Such biomarkers could be instrumental in enabling timely interventions, potentially reducing the adverse effects of these conditions on fetal brain development. Ultimately, this work lays the foundation for future research exploring the mechanisms by which pregnancy diseases impact the developing brain and the potential for therapeutic strategies to mitigate these effects.

## 3. Materials and Methods

All animal experiments were approved by the Institutional Animal Care and Use Committee (IACUC) at the University of Missouri–Kansas City. Mice were housed in a controlled environment with regulated temperature and humidity, on a 12 h light–dark cycle. C57BL/6J male and female mice, aged 8–12 weeks, were initially obtained from the Jackson Laboratory (Bar Harbor, ME, USA) and subsequently bred in-house to provide the required males and females for this study. Trio breeding was employed, with pregnancies confirmed by the detection of a vaginal plug. The day of detection of the plug was considered as gestational day (GD) 1. At least 3–5 females from each gestational group were euthanized, as described previously [42,43], and a minimum of five embryos from each female were collected on GD 10, 12, 15, and 18 for further analysis.

Frozen brain tissue sections from various gestational stages were prepared as previously described [42,43]. In brief, tissues were fixed in 4% paraformaldehyde (PFA) in PBS for 4 h at 4 °C, then processed through a 10–30% sucrose gradient at 4 °C and embedded in Optimal Cutting Temperature (OCT) compound. Frozen blocks were prepared in 2-methylbutane in liquid nitrogen and stored at −80 °C until further use. Immunostaining (IHC) was performed in 10 μm frozen sections fixed with 4% PFA in PBS for 10 min. The sections were then incubated with blocking serum, followed by overnight incubation at 4 °C with commercially available primary antibodies. Detection was performed using biotinylated secondary antibodies, all of which have been extensively validated in our laboratory [42,43,44]. Primary antibodies used included NG2 (1:500; rabbit polyclonal; Chemicon, Temecula, CA, USA) for pericyte detection, CD31 (1:200; rat monoclonal; BD Pharmingen, San Diego, CA, USA) for early-stage endothelial cells involved in vessel formation, von Willebrand Factor (vWF; 1:4000; Dako Corporation, Carpinteria, CA, USA) for mature vessels, Glial Fibrillar Acidic Protein (GFAP; mouse monoclonal, M0761) for astrocytes, and claudin-5 (mouse monoclonal, 352588) for tight junctions. Specimens were visualized using either a Vectastain kit for diaminobenzidine (DAB) staining or fluorescein-labeled avidin (Vector Laboratories, Burlingame, CA, USA), based on the recommendations of the primary antibody manufacturer and the sensitivity requirements of the assay system. For immunofluorescence analysis, sections were mounted with Vectashield containing 4′,6′-diamidino-2-phenylindole (DAPI; Vector Laboratories, Burlingame, CA, USA). Imaging was performed using a Zeiss Axioskop 2 microscope equipped with a Zeiss AxioCam camera for both fluorescence and bright-field imaging (Carl Zeiss, Oberkochen, Germany).

## Data Availability

Data are included in the article, and further data are available from the investigators and will be provided upon request.

## Abbreviations

The following abbreviations are used in this manuscript:

BBB	Blood–brain barrier
GD	Gestational day
FGR	Fetal growth restriction
PNVP	Perineural vascular plexus
SVP	Subventricular vascular plexus
